# Arsinothricin, an arsenic-containing non-proteinogenic amino acid analog of glutamate, is a broad-spectrum antibiotic

**DOI:** 10.1038/s42003-019-0365-y

**Published:** 2019-04-15

**Authors:** Venkadesh Sarkarai Nadar, Jian Chen, Dharmendra S. Dheeman, Adriana Emilce Galván, Kunie Yoshinaga-Sakurai, Palani Kandavelu, Banumathi Sankaran, Masato Kuramata, Satoru Ishikawa, Barry P. Rosen, Masafumi Yoshinaga

**Affiliations:** 1 Department of Cellular Biology and Pharmacology, Florida International University, Herbert Wertheim College of Medicine, Miami, FL 33199 USA; 2 Planta Piloto de Procesos Industriales Microbiológicos (PROIMI-CONICET), Tucumán, T4001MVB Argentina; 3 SER-CAT and Department of Biochemistry and Molecular Biology, University of Georgia, Athens, GA 30602 USA; 4 Berkeley Center for Structural Biology, Lawrence Berkeley Laboratory, Berkeley, CA 94720 USA; 5 Division of Hazardous Chemicals, National Institute for Agro-Environmental Sciences, NARO, Tsukuba, Ibaraki, 305-8604 Japan; 6 Present Address: Manchester Institute of Biotechnology, School of Chemistry, University of Manchester, 131 Princess Street, Manchester, M1 7DN UK

## Abstract

The emergence and spread of antimicrobial resistance highlights the urgent need for new antibiotics. Organoarsenicals have been used as antimicrobials since Paul Ehrlich’s salvarsan. Recently a soil bacterium was shown to produce the organoarsenical arsinothricin. We demonstrate that arsinothricin, a non-proteinogenic analog of glutamate that inhibits glutamine synthetase, is an effective broad-spectrum antibiotic against both Gram-positive and Gram-negative bacteria, suggesting that bacteria have evolved the ability to utilize the pervasive environmental toxic metalloid arsenic to produce a potent antimicrobial. With every new antibiotic, resistance inevitably arises. The *arsN1* gene, widely distributed in bacterial arsenic resistance (*ars*) operons, selectively confers resistance to arsinothricin by acetylation of the α-amino group. Crystal structures of ArsN1 *N*-acetyltransferase, with or without arsinothricin, shed light on the mechanism of its substrate selectivity. These findings have the potential for development of a new class of organoarsenical antimicrobials and ArsN1 inhibitors.

## Abstract (web summary)

Nadar, Chen, Dheeman et al. show that arsinothricin, an arsenic-containing non- proteinogenic amino acid analog of glutamate, is an effective broad-spectrum antibiotic through inhibition of glutamine synthetase. This study suggests a possibility of developing a new class of antimicrobials that thwart microbial resistance to arsinothricin.

## Introduction

Arsenic is the most pervasive environmental toxic element^1^. Here we describe how bacteria harness arsenic to create a potent broad-spectrum antibiotic. New antibiotics are urgently needed because the emergence of resistance has rendered nearly every clinically used antibiotic ineffectual. Human tuberculosis, the top global infectious disease killer, which is caused by *Mycobacterium tuberculosis*, has become even more difficult to treat due to the drug resistance^2^. The World Health Organization declares multidrug-resistant tuberculosis a global public health crisis, calling for a pressing need for development of new and innovative antibiotics^3^. In addition to *M. tuberculosis*, the World Health Organization recently issued a global priority pathogen list of antibiotic-resistant bacteria that pose the greatest threat to human health to guide and promote research and development of new antibiotics^4^.

The use of arsenicals as antimicrobial and anticancer agents is well-established^5,6^. The first synthetic antimicrobial agents were the organoarsenicals atoxyl (*p*-aminophenylarsenate, also known as *p*-arsanilic acid) and salvarsan (arsphenamine). While salvarsan is no longer in clinical use, the organoarsenical melarsoprol, developed in 1949, is still recommended by the World Health Organization for treatment of second-stage *Trypanosoma brucei* sleeping sickness^7^. Atoxyl and the related synthetic aromatic arsenicals roxarsone (4-hydroxy-3-nitrophenylarsenate) and nitarsone (4-nitrophenylarsenate) are antimicrobials used for the prevention of *Coccidia* and *Histomonas* infections in poultry^8^. Although no longer in wide use in the United States, roxarsone is still produced and utilized worldwide. Finally, arsenic trioxide is currently the treatment of choice in humans for all-trans retinoic acid unresponsive acute promyelocytic anemia^9^.

**Fig. 1:**
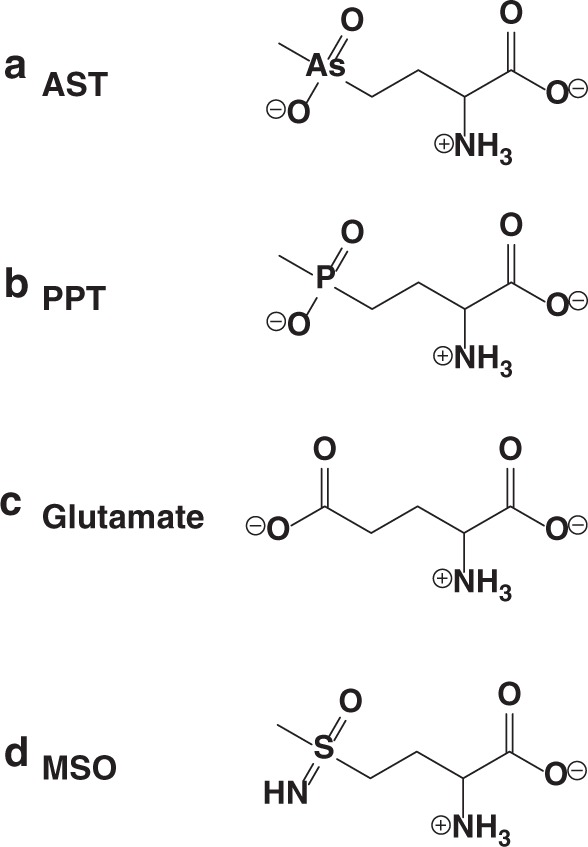
Chemical structure of glutamate and analogs. **a** Arsinothricin (AST); **b** phosphinothricin (PPT); **c** glutamate; **d** methionine sulfoximine (MSO)

Here we demonstrate that a recently discovered arsenic-containing natural product, arsinothricin (2-amino-4-(hydroxymethylarsinoyl)butanoate, AST) (Fig. 1a), produced by the rice rhizosphere microbe *Burkholderia gladioli* GSRB05^10^, has broad-spectrum antibiotic activity. Biosynthetic AST is a mimetic of the *Streptomyces* antibiotic L-phosphinothricin (2-amino-4-(hydroxymethylphosphinyl)butanoate or L-PPT) with an arsenic in place of the phosphorus of L-PPT (Fig. 1b). L-AST and L-PPT are non-proteinogenic amino acid analogs of l-glutamate (Fig. 1c) and act through inhibition of glutamine synthetase. Most toxic arsenicals contain trivalent As(III). AST is unusual in being a highly toxic pentavalent organoarsenical. It is chemically unrelated to other organoarsenicals and has the potential to be the progenitor of a new class of organoarsenical antibiotics. With every new antibiotic, resistance inevitably arises. The enzyme PPT *N*-acetyltransferase (PAT) confers resistance to PPT by acetylating its α-amino group. A curious observation has been that many arsenic resistance (*ars*) operons have an *arsN1* gene that encodes a *pat* ortholog. Why an enzyme for PPT resistance should be in an *ars* operon was a mystery. The identification of AST as a natural product suggested that the biological function of ArsN could be to act as an AST resistance. Here we show that ArsN1 acetylates both AST and PPT but with higher affinity for AST, indicating that ArsN1 is an AST-selective *N*-acetyltransferase. We crystallized ArsN1 and solved the apo and substrate-bound structures. This knowledge can be utilized to design new and novel drugs that evade or inhibit resistance mechanisms.

## Results

### AST is a broad-spectrum antibiotic

**Fig. 2:**
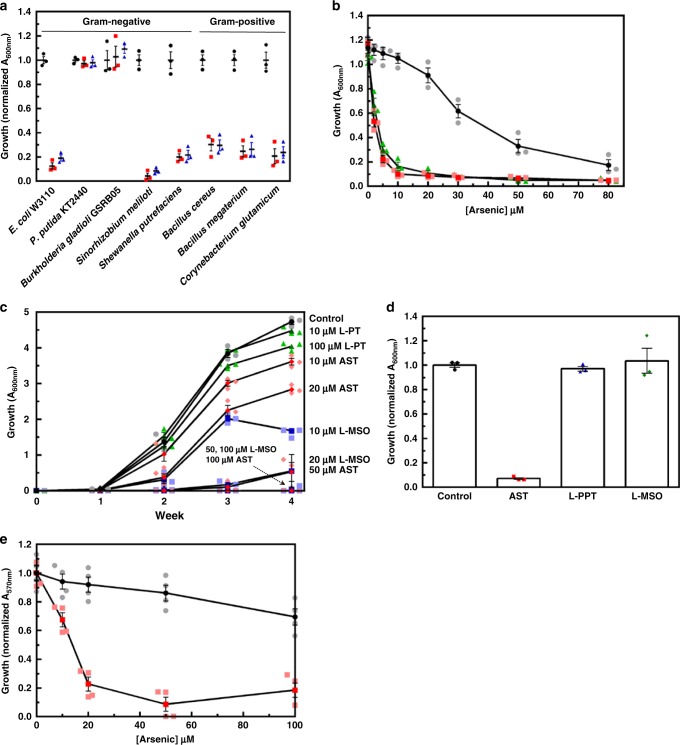
AST is a broad-spectrum antibiotic. **a** AST inhibits growth of both Gram-negative and Gram-positive bacteria. Strains were cultured in M9 medium in the absence (black circles) or presence of 25 µM AST (red squares) or 400 µM L-PPT (blue triangles) as described in Methods, and growth was estimated from the *A*_600nm_ after 24 h. Data are the mean ± SE (*n* = 3). **b** Pentavalent AST is more toxic than trivalent As(III). The toxicity of AST (triangles) was compared with MAs(III) (squares) and As(III) (circles) in *E. coli* AW3110 grown in M9 medium. Growth was estimated from A_600nm_ after 24 h. Data are the mean ± SE (*n* = 3). Dark- and light-colored symbols represent means and individual data points, respectively. **c** Effect of AST on mycobacterial growth. Cultures of *M. bovis* BCG were inoculated at an initial density of 10^5^ cells/ml and then incubated at 37 °C in a 5% CO_2_ atmosphere for up to 4 weeks in the absence (Control, circles) or presence of the indicated concentrations of GS inhibitors L-MSO (squares), L-PPT (triangles) or AST (diamonds). Growth was estimated from A_600nm_. Data are the mean ± SE (*n* = 3). Dark- and light-colored symbols represent means and individual data points, respectively. **d** Effect of AST on carbapenem-resistant *E*. *cloacae*. Cells were cultured in M9 medium in the absence (Control) or presence of 25 µM AST, L-PPT or L-MSO, with growth estimated from the A_600nm_ after 24 h. Data are the mean ± SE (*n* = 3). **e** Cytotoxicity of AST in human monocytes. Human THP-1 cells were incubated in the presence or absence of the indicated concentrations of As(III) (circles) or AST (squares) for 24 h, and viability was determined using a 3-(4,5-dimethylthiazol-2-yl) 2,5-diphenyltetrazolium bromide assay, as described in Methods. Data are the mean ± SE (*n* = 4). Dark- and light-colored symbols represent means and individual data points, respectively

To determine whether AST has antibiotic activity, we examined its ability to inhibit growth of bacteria using environmental isolates. AST was equally effective against both Gram-negative and Gram-positive bacteria (Fig. 2a). Each species was inhibited to the same degree by 25 μM AST and 400 μM L-PPT, except for *B. gladioli* GSRB05 and *Pseudomonas putida* KT2440. *B. gladioli* GSRB05 is the producer of AST^10^, so it is not unexpected that this strain might be resistant to the antibiotic it produces. As discussed below, the *arsN1* gene confers resistance in *P. putida* KT2440. Our results demonstrate that AST is a broad-spectrum antibiotic effective against both Gram-negative and Gram-positive bacteria. In *Escherichia coli*, AST is considerably more inhibitory than inorganic As(III) and is similar to that of highly toxic trivalent methylarsenite (MAs(III)) (Fig. 2b). Given that, in general, pentavalent arsenicals are relatively benign and much less toxic compared to trivalent species^11^, this is a striking result. To our best knowledge, except thiolated species^6^, AST is the only known pentavalent arsenic species that exhibits high toxicity.

### AST inhibits glutamine synthetase

The mechanism of action of L-PPT is irreversible inhibition of bacterial glutamine synthetase^12^. L-PPT also inhibits plant glutamine synthetase, which is the basis for its use as the broad-spectrum systemic herbicide Glufosinate^13^. Because of the structural similarity with PPT (Fig. 1b), it was reasonable to propose that the target of AST is also bacterial glutamine synthetase. We compared the effect of AST and L-PPT on purified *E. coli* glutamine synthetase activity. The *K*_m_ of glutamine synthetase was found to be 2.7 ± 0.6 mM for l-glutamate, consistent with the previous determination^12^. The observed *K*_i_ values for AST and L-PPT are 0.3 ± 0.05 μM and 0.4 ± 0.15 μM, respectively, indicating that AST is as effective an inhibitor of glutamine synthetase as is L-PPT.

### AST is an effective antibiotic with pathogenic bacteria

Inhibition of glutamine synthetase has been proposed to be a potential therapeutic strategy against tuberculosis^14^. Pathogenic mycobacteria, including *M. tuberculosis*, secrete large amounts of an extracellular glutamine synthetase that is involved in synthesis of the poly-α-l-glutamine layer, a cell wall component that is found exclusively in pathogenic strains and considered essential to their virulence^15^. In fact, L-methionine *S*-sulfoximine (L-MSO) (Fig. 1d), the first glutamine synthetase inhibitor described^16^, effectively inhibits *M. tuberculosis* growth both in vitro and in vivo^15,17^. To examine the potential of AST as a drug for tuberculosis, we analyzed the effect of AST on a related pathogenic strain, *M. bovis* BCG, and compared it with L-PPT and L-MSO (Fig. 2c). AST inhibits mycobacterial growth at concentrations comparable to L-MSO and is a much better inhibitor than L-PPT. AST also effectively inhibits growth of carbapenem-resistant *Enterobacter cloacae* (ATCC BAA-2341), which belongs to the highest priority category in the World Health Organization global priority pathogens list^3^, whereas other glutamine synthetase inhibitors have no effect on growth of *E. cloacae* (Fig. 2d). Among the glutamine synthetase inhibitors examined, only AST effectively inhibits growth of both *Mycobacterium* and *Enterobacter* pathogens, which strongly suggests that AST is a useful lead compound for potential development of new antimicrobial drugs against antibiotic-resistant pathogens. Importantly, AST is much less cytotoxic to human monocytes compared with inorganic arsenite (Fig. 2e). This low cytotoxicity further supports the potential of AST as a lead compound for drug development.

### PpArsN1 confers resistance to arsinothricin

**Fig. 3:**
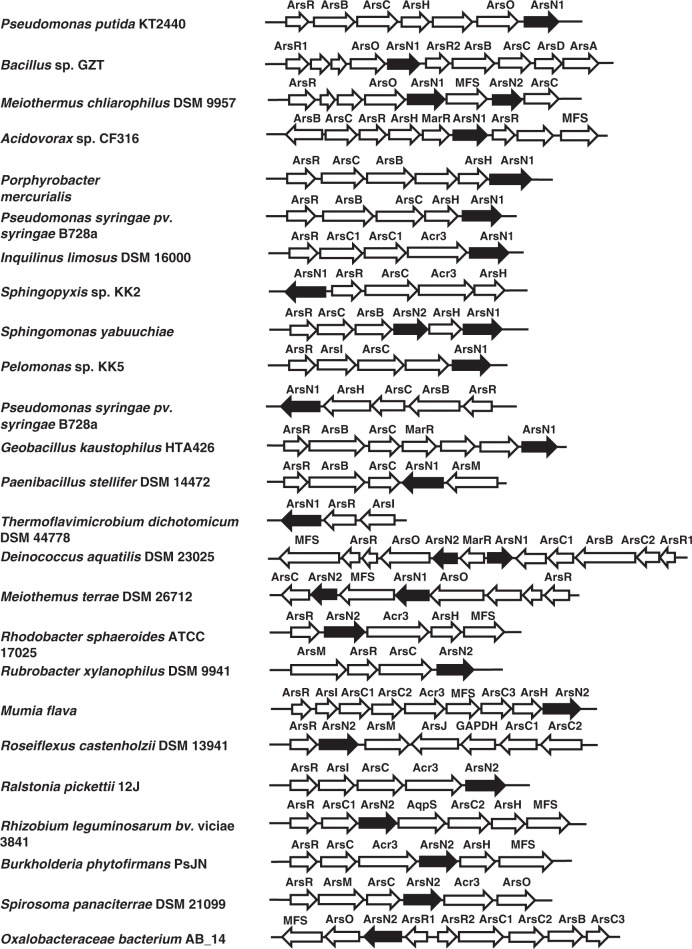
Compilation of bacterial *ars* operons with *arsN* genes. Shown are representative *ars* operons containing *arsN* genes (black fill). GenBank accession numbers are given in “Methods” section

**Fig. 4:**
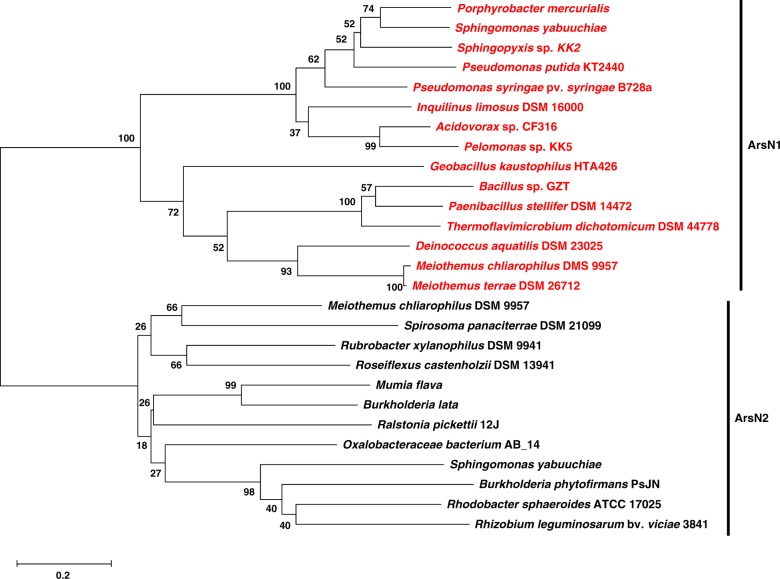
Phylogeny of *arsN* genes. The neighbor-joining phylogenetic tree shows evolutionary relationships. All *arsN* genes are located in *ars* operons. The genes are sorted into two clades, *arsN1* (highlighted in red) and *arsN2*. Bootstrap values calculated for 1,000 subsets (%) are indicated on each branch. GenBank accession numbers of bacterial genomes are given in Methods. The scale bar represents 20% sequence dissimilarity

**Fig. 5:**
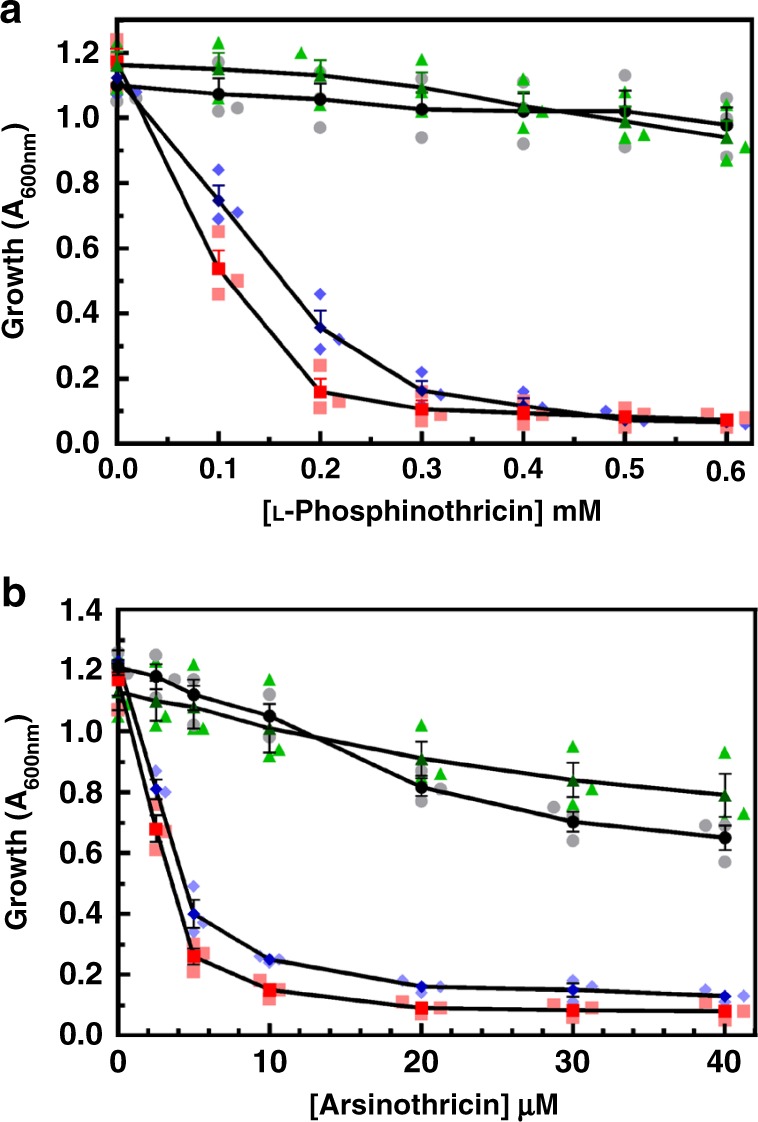
**a** PpArsN1 confers resistance to L-PPT (**a**) and AST (**b**). **a** Strains: wild type *P. putida* (circles); *Δars1,2* (squares); *E. coli* AW3110 bearing vector plasmid (diamonds) or plasmid carrying *PparsN1* (triangles). Cells were cultured in M9 medium with the indicated concentrations of L-PPT. Growth was estimated from *A*_600nm_ after 24 h. Data are the mean ± SE (*n* = 3). Dark- and light-colored symbols represent means and individual data points, respectively. **b** Cells were treated with the indicated concentrations of AST as in **a**. Data are the mean ± SE (*n* = 3). Dark- and light-colored symbols represent means and individual data points, respectively

Bacterial resistance to L-PPT is conferred by phosphinothricin *N*-acetyltransferases (PATs)^18^. These inactivate L-PPT by acetylation of the α-amino group, which prevents binding to glutamine synthetase^12^. These genes have been used to construct transgenic PPT-resistant plants, allowing D,L-PPT to be used for weed control^13^. Many bacterial *ars* operons have genes that encode putative GCN5-related *N*-acetyltransferases^19^ (Fig. 3). These genes can be sorted into two clades (Fig. 4). The genes in Clade 1 encode proteins more closely related to phosphinothricin *N*-acetyltransferases, whereas products of the genes from Clade 2 are more closely related to glutamate *N*-acetyltransferases (*N*-acetylglutamate synthases)^20^. We term the former *arsN1* and the latter *arsN2*. In this report we focus on *arsN1*. The *arsN1* gene of *P. putida* KT2440 (*PparsN1*, accession number AAN67541) was originally termed *phoN1* because it was shown to confer L-PPT resistance^21^. The genome of *P. putida* KT2440 has two *ars* operons (*ars1* and *ars2*), and the *ars1* operon contains the *PparsN1* gene. Wild type cells are L-PPT resistance, while cells with a deletion of both *ars* operons (Δars1,2) are sensitive to L-PPT (Fig. 5a). Introduction of *PparsN1* into *E. coli* AW3110 confers L-PPT resistance, which was consistent with previous studies^21^.

To date every characterized *ars* gene has been shown to have an arsenic-related function, so it is unlikely that the primary function of PpArsN1 is PPT resistance because PPT does not contain arsenic. The prevalence of *arsN* genes in multiple *ars* operons implies involvement in arsenic metabolism. It was logical to propose that AST is the primary substrate of ArsN1. Parental *P. putida* is resistant to AST, while *P. putida Δars1,2* is sensitive (Fig. 5b). *E. coli* AW3110 is similarly sensitive to AST, and heterologous expression of *PparsN1* confers resistance. These results support our hypothesis that ArsN1 has the arsenic-related function of AST resistance. Comparing the effect of L-PPT with AST, both *P. putida Δars1,2* and *E. coli* AW3110 show nearly complete inhibition of growth by 20 μM AST, with 50% inhibition at ~3 μM AST. In contrast, 100 μM L-PPT was required to give 50% inhibition. The result indicates that AST is at least 30-fold more effective as an antibiotic compared with L-PPT.

### PpArsN1 is an arsinothricin-selective *N*-acetyltransferase

Crude extracts of cells of *P. putida* expressing *PparsN1* have been shown to acetylate PPT^21^. Here we demonstrate that purified PpArsN1 exhibits phosphinothricin acetyltransferase activity (Table 1). The glutamine synthetase inhibitor L-MSO is a poorer substrate compared with AST (Fig. 1d). Purified PpArsN1 has 100-fold higher affinity for AST compared with L-PPT, and 15-fold higher catalytic efficiency (*K*_cat_/*K*_m_), indicating that AST is the physiological substrate of PpArsN1. The affinity and catalytic efficiency of *Streptomyces viridochromogenes* phosphinothricin *N*-acetyltransferase (SvPAT) with AST are similar with those of PpArsN1. In contrast, SvPAT shows two orders of magnitude higher affinity for PPT than PpArsN1. Thus, while ArsN1 is selective for AST, SvPAT has similar affinity for both PPT and AST.

**Table 1:** PpArsN1 is selective for L-AST over other glutamine synthetase inhibitors

Substrate (50 μM)	Specific activity (nmol s^−1^ mg^−1^ PpArsN1)			
AST	49.6 ± 0.8			
L-PPT	13.9 ± 1.9			
L-MSO	2.1 ± 0.1			

Enzyme	Substrate	*K*_m_ (μM)	*K*_cat_ (s^−1^)	*K*_cat_/*K*_m_ (M^−1^ s^−1^)
PpArsN1	AST	11 ± 3	1.7 ± 0.2	1.55 × 10^5^
	L-PPT	1000 ± 200	9.6 ± 0.9	0.10 × 10^5^
SvPAT	AST	12 ± 2	2.3 ± 0.1	1.92 × 10^5^
	L-PPT	47 ± 2	3.1 ± 0.0	0.66 × 10^5^

### Crystal structure of PpArsN1

**Fig. 6:**
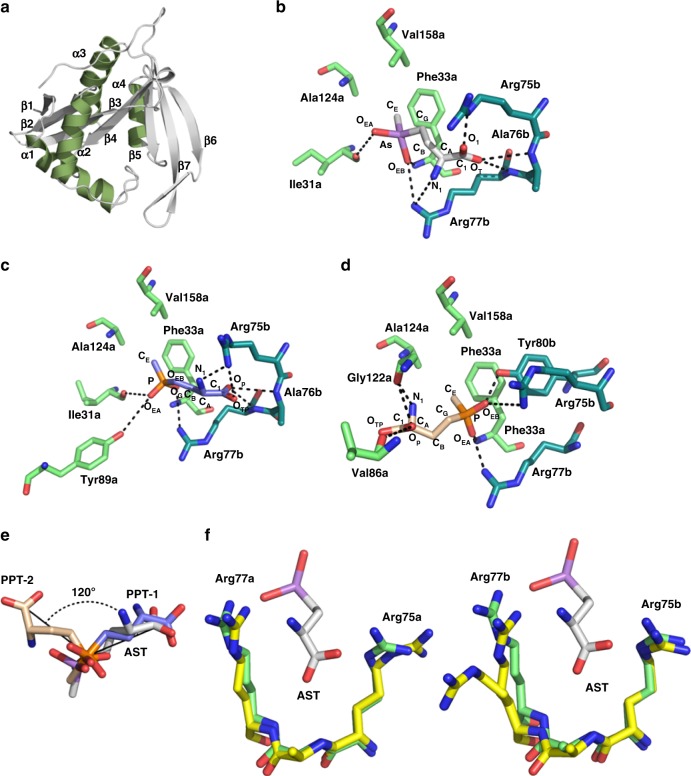
Structure of PpArsN1. **a** Overall fold of apo-PpArsN1. α helices are shown in green. Helices α1, α2 and α3, α4 are in the outer layers, and the seven β strands are in the inner layer of the sandwich. There is a structurally-conserved β bulge in the middle of the strands. **b** Interaction of L-AST with PpArsN1. The AST binding site is formed by residues from both chains. Arg75b, Ala76b and Arg77b (teal) of Chain B project into the AST binding site of Chain A, Ile31a, Phe33a, Ala124a and Val158a (green). Distances between polar atoms are less than 4.0 Å (dotted lines). C_E_ of L-AST is surrounded by Phe33a, Ala124a and Val158a. O_EA_ and O_EB_ of L-AST interact with Ile31a and with Arg77b and Phe33a, respectively. O_1_ and O_T_ of L-AST interact with Arg75b and with Arg77b and Ala76b, respectively. N_1_ of AST coordinates with Arg77b. **c** Interaction of PPT-1 (conformation 1) with PpArsN1. PPT-1 interacts with PpArsN1 in a way similar to AST with additional interactions: 1) the O_EA_ atom with Ile31a and Tyr89a; and 2) the amino group with Arg75b. **d** Interaction of PPT-2 (conformation 2) with PpArsN1. C_E_ of PPT is surrounded by Phe33a, Ala124a and Val158a. The atom O_EA_ interacts with Arg77b. The O_EB_ atom interacts with Arg75b, Tyr80b and Phe33a. The atom O_P_ interacts with Val86a and Gly122a. The amino group interacts with Gly122a. **e** Orientation of L-AST and L-PPT. When the As atom of L-AST and the P atoms of PPT-1 and PPT-2 are superimposed, the carboxylates of PPT-2 and L-AST are oriented 120° relative to each other, and the carboxylates of PPT-1 and AST are oriented in the same direction. **f** A conformational change of PpArsN1 resulting from ligand binding. A portion of the AST binding site in PpArsN1-AST (green) is superimposed with that of the apo structure (yellow). Left and right cartoons depict Chain A and Chain B, respectively. Arg75a in Chain A and Arg77b in Chain B are closer to L-AST when substrate is bound

To elucidate the mechanism of PpArsN1 resistance and its selectivity for AST, we solved the structure of apo- and substrate-bound PpArsN1. The overall conformation is a three-layer α/β sandwich fold (Fig. 6a), a typical GCN5-related *N*-acetyltransferase fold^22^. PpArsN1 forms an asymmetric homodimer in solution, as shown by the extensive interactions of the subunits (Supplementary Figure 1) and size-exclusion chromatography (Supplementary Figure 2), similar to related *N*-acetyltransferases. The AST-bound PpArsN structure shows that the L-enantiomer is the substrate of the *N*-acetyltransferase, which supports our assumption that L-AST is the active form of the antibiotic (Supplementary Figure 3). PpArsN1 has two L-AST-binding sites, which are asymmetrically formed by amino acid residues from both Chains A and B. Both binding sites are composed of seven residues: four residues from Chain A (Ile31a, Phe33a, Ala124a and Val158a) (Fig. 6b, green) and three residues from Chain B (Arg75b, Ala76b and Arg77b) (Fig. 6b, teal). L-PPT is bound in two conformations. In one conformation (PPT-1) (Fig. 6c), the orientation of L-PPT is similar to that of L-AST (Fig. 6b), although the sets of amino acid residues used by PpArsN1 to interact with each chemical moiety in L-PPT are slightly different from those that interact with the corresponding chemical moiety in L-AST. In these structures, the predicted distance between the α-amino group of AST/PPT and the sulfur atom of acetyl coenzyme A (AcCoA) is too long to initiate acetylation (Supplementary Figure 4). Another conformation of L-PPT (PPT-2) (Fig. 6d) is similar to that of L-PPT observed in the previously reported coenzyme A- and L-PPT-bound ShPAT (PAT from *Streptomyces hygroscopicus*, also known as BAR)^23^. Superimposition of these two conformations of L-PPT-bound PpArsN1 demonstrates the two different binding modes of substrates in PpArsN1 (Fig. 6e). The arsenic atom of L-AST closely overlaps the phosphorus atom in PPT-1 and PPT-2. The orientation of PPT-1 is almost superimposable with that of L-AST. In contrast, the orientation of PPT-2 is inclined at 120° towards the AcCoA binding site with respect to those of PPT-1 and L-AST. This brings the α-amino group of L-PPT closer to the sulfur atom of AcCoA (Supplementary Figure 4), which is more favorable for catalysis. Arg75a in Chain A of the apo-structure also shows two conformations. One superimposes with the L-AST-bound PpArsN1 structure, covering the substrate-binding channel, whereas the other moves out of channel (Fig. 6f). The side chain of Arg77b in Chain B also appears to cover and move away from the substrate-binding site, allowing substrate access to the active site. The two conformations of this residue in L-PPT-bound PpArsN1, for both PPT-1 and PPT-2, are quite similar to those in L-AST-bound PpArsN1 (Supplementary Figure 5). Arg75 and Arg77 from each subunit appear to form gates that controls substrate access to both catalytic sites.

## Discussion

Arsenic is the most ubiquitous environmental poison, and its toxicity presented a challenge to the first organisms^1^. Arsenic is and always has been the most prevalent toxic substance in surface and subsurface waters and soil. To adapt to high arsenic concentrations in primordial waters, microbes evolved arsenic detoxification mechanisms more than 2.5 billion years ago^24^. In addition, microbes developed mechanisms to use arsenic for energy production^25^. So it is not unexpected that bacteria would evolve pathways to use arsenic as an antibiotic to give them a selective growth advantage over competitors^26^. Here we identify the organoarsenical AST as a novel natural product with broad-spectrum antibiotic properties synthesized by an environmental isolate of *Burkhoderia*. Although the pathway of AST synthesis is not known, the biosynthetic pathway of phosphinothricin consists of more than twenty genes^27^, which suggests that the pathway for AST synthesis will prove to be correspondingly complicated.

As Paul Ehrlich, who synthesized the antimicrobial organoarsenical salvarsan, predicted, drug resistance follows the drug like a faithful shadow^28^. The *arsN1* gene encodes an *N*-acetyltransferase that confers resistance to arsinothricin with high selectivity over the related antibiotic phosphinothricin. The *arsN1* gene is present in many species of soil bacteria, which implies that AST is synthesized by other members of microbial communities, and that AST will be found to be present in soil and water with even moderate concentrations of arsenic. Indeed, we predict that the extensive distribution of *arsN1* genes reflects an equally wide occurrence of AST producers, an eminently testable hypothesis.

We propose that the small difference in the As–O and P–O bond lengths allows L-AST to bind more tightly to PpArsN1 than L-PPT. Known differences between arsenic and phosphorus coordination are instructive. In arsenate the As–O bond length is 1.69 Å compared with a P–O bond length of 1.52 Å in phosphate^29^. In the periplasmic phosphate binding protein of *Halomonas* sp. GFAJ-1 this minute difference in bond length distorts a low-barrier H-bond and allows a 4500-fold selectivity for phosphate over arsenate^30^. L-AST and L-PPT differ from inorganic arsenate and phosphate in having C-As and C-P bonds replacing O–As and O–P bonds. In L-AST-bound PpArsN1, the bond lengths of As–C_G_, As–C_E_, As–O_EA_, As–O_EB_ are 2.0, 1.9, 1.9 and 2.0 Å, respectively. In L-PPT, the bond lengths of P–C_G_, P–C_E_, P–O_EA_ and P–O_EB_ are 1.8, 1.8, 1.6 and 1.5 Å, respectively. Although small, these differences are critical for binding affinity. Both the arsenic atom in L-AST and the phosphorus atom in L-PPT are in a tetrahedral geometry with four coordinations. The volume of the L-AST tetrahedron is 3.00 Å^3^, compared with 1.86 Å^3^ for L-PPT. We predict that this substantial difference in substrate volume affects hydrogen bonding, hydrophobic and van der Waal contacts between the tetrahedral substrates and enzyme that accounts in part for the 100-fold higher affinity of ArsN1 for L-AST compared with L-PPT.

The high selectivity of PpArsN1 for AST suggests that *arsN1* genes evolved in response to the environmental challenge presented by AST producers. The results of phylogenetic analysis suggest that ArsN1 genes can be further sorted into two subclades, with PAT and MAT branching off from both ArsN1 subclades (Supplementary Figure 6). This implies that ArsN1 is the common ancestor of those *N*-acetyltransferase members and that the arsenical antibiotic AST is the most ancient of this class of antimicrobials. Given that SvPAT has similar affinity for both PPT and AST (Table 1), we speculate that PAT homologs evolved from ArsN1 to increase affinity for PPT in response to PPT emergence without losing affinity for AST. The ten residues involved in L-PPT binding in ShPAT are conserved in SvPAT, while three are conservatively replaced in PpArsN1 (Supplementary Figure 7). These minor differences in amino acid residues involved in substrate-binding between ArsN1 and PAT may lead in part to the differences in their substrate selectivity. AST was only recently identified, so we might predict that other organoarsenical antibiotics exist. There are genes in *ars* operons for which functions have not been found; these might be resistance mechanisms against unknown natural products containing arsenic.

One concern is that ArsN1 resistance to AST could be a limiting factor for future clinical use. However, the effectiveness of AST could be extended if it could be used in combination with ArsN1 inhibitors^31,32^. To this end, analysis of the structure of ArsN1 is enlightening. Given the shorter distance between the amino group of PPT and the acetyl group of AcCoA in the PPT-2 conformation compared with the longer distance in the PPT-1 conformation (Supplementary Figure 4), it is reasonable to propose that PPT-2, and not PPT-1, is a conformation that the enzyme assumes during catalysis. The logical next question is why L-PPT binds to PpArsN1 in both the PPT-1 and the PPT-2 conformations. The α-amino group of L-PPT must be deprotonated for acetylation to occur. In ShPAT, like the other GCN5-related *N*-acetyltransferases, a conserved catalytic Glu88 acts as a general base, interacting with the α-amino group of L-PPT via a water molecule. The enzyme then uses the water molecule as the proton shuttle to catalyze the deprotonation step^23^. In PpArsN1, however, the residue corresponding to Glu88 of ShPAT is Asp85 (Supplementary Figure 7). The side chain of Asp85 is not long enough to catalyze deprotonation of the α-amino group of L-PPT in the PPT-2 conformation. In that conformation, no water molecule interactions were found between the α-amino group of L-PPT and Asp85 of PpArsN1 (Supplementary Figure 8a, 9.7 Å). In contrast, in the PPT-1 conformation, the distance between the α-amino group of L-PPT and Asp85 is shorter (Supplementary Figure 8a, 4.9 Å), allowing Asp85 to form a coordination with the amino group of L-PPT via a water molecule. This suggests that substrate deprotonation is catalyzed by Asp85 in the PPT-1 conformation. A similar water molecule bridge was also observed between AST and Asp85 in AST-bound PpArsN1 (Supplementary Figure 8b). Based on these results, we propose that PpArsN1 has two separate sites for substrate deprotonation and acetylation. In this hypothesis, PpArsN1 first captures the substrate in the deprotonation site, as seen in the PPT-1 conformation (Fig. 6c), where the α-amino group of the substrate is deprotonated by Asp85 (Supplementary Figure 8a). The deprotonated substrate then relocates to the acetylation site, as seen in the PPT-2 conformation (Fig. 6d), where the distance between the deprotonated substrate to AcCoA is shorter (Supplementary Figure 4). This allows nucleophilic attack on the carbonyl bond of the acetyl group, promoting catalysis. Based on structural analysis, a similar mechanism that uses separate sites for deprotonation and acetylation of substrate has been proposed for l-glutamate *N*-acetyltransferase from *M. tuberculosis*^33^. A unique feature that differentiates PpArsN1 from ShPAT is that the latter utilizes a common site for both deprotonation and acetylation of the substrate^23^.

With such knowledge obtained through our study, it may be possible to develop ArsN1 inhibitors that can be used in combination with AST to prevent resistance. In addition, from the structure of the binding site, we can rationally propose syntheses of more potent AST derivatives that bind to glutamine synthetase with higher affinity or bind to ArsN1 with lower affinity. In summary, we predict that AST may be the progenitor of a new class of antibiotics.

## Methods

### Reagents

All reagents and enzymes were purchased from Sigma-Aldrich Co. LLC (St. Louis, MO, USA), unless otherwise stated. Arsinothricin (AST) was purified from cultures of *B. gladioli* GSRB05, as described previously^10^. The concentration and purity of purified AST were determined by inductively coupled plasma mass spectrometry (ELAN DRC-e; Perkin–Elmer, Waltham, MA, USA) and high pressure liquid chromatography (series 2000, Perkin–Elmer) coupled to inductively coupled plasma mass spectrometry. AST is assumed to be the L-enantiomeric form based on the ArsN1 crystal structure with bound L-AST (*vide infra*). Commercial phosphinothricin (PPT) and methionine *S*-sulfoximine (MSO) are the D,L- and L-enantiomers, respectively. In the studies described below the concentration of D,L-PPT was divided by a factor of 2 to give the concentration of the L-enantiomer, the active form of the antibiotic^13^. Methylarsonous acid (MAs(III)) was prepared as described previously^34^.

### Bacterial strains

*Escherichia coli* strains DH5α (Promega, Madison, WI, USA) and TOP10 (Invitrogen, Waltham, MA, USA) were used for gene cloning and protein expression, respectively. *E. coli* strain W3110^35^ and the *ars* operon deleted derivative AW3110 (*Δars*)^36^, *Pseudomonas putida* KT2440 and the double *ars* operon deleted derivative strain (*Δars1,2*)^21^, *Burkholderia gladioli* GSRB05^9^, *Shinorhizobium meliloti* Rm1021^37^, *Shewanella putrefaciens* 200, *Bacillus cereus* UW85^38^, *Bacillus megaterium* (ATCC 14581), *Corynebacterium glutamicum* (ATCC 13032), *Enterobacter cloacae* (ATCC BAA-2341) and *Mycobacterium bovis* BCG (ATCC 19274) were used for in vivo resistance assay.

### Cloning, expression and protein purification

For gene cloning and protein expression, *E. coli* cells were grown at 37 °C in lysogeny broth (LB) medium^39^ supplemented with 0.1 mg/ml ampicillin. For construction of a plasmid for expression of *arsN1* from *P. putida* KT2440 (*PparsN1*) (accession number: AAN67541.1) in fusion with a six histidine tag at C-terminus, a 558-bp fragment excluding the stop codon was PCR-amplified from total genomic DNA of *P. putida* KT2440 by high fidelity *PfuTurbo* DNA polymerase (Agilent Technologies Inc., Santa Clara, CA, USA) using the forward primer 5′-CCAGCCATGGATAGCGGAATCGATATTCG-3′ (*Nco*I site underlined) and reverse primer 5′-CCAGAAGCTTACGAGGCACTGGGATTTGG-3′ (*Hind*III site underlined) and then ligated into pBAD-Myc/His-A as an *Nco*I/*Hind*III digest, generating the plasmid pBAD-*PparsN1*. The DNA sequence for *pat*, the gene encoding phosphinothricin *N*-acetyltransferase from *Streptomyces viridochromogenes* (*Svpat*) (accession number: AAU00088.1) with six histidine codons inserted at the 3’ end before the stop codon, was chemically synthesized by GenScript (NJ, USA) with 5′ *Nco*I and 3’ *Hind*III sites and cloned into the *EcoR*V site of pUC57-Kan (pUC57-Kan-*Svpat*). The synthetic *Svpat* gene was cloned as an *Nco*I/*Hind*III digest from pUC57-Kan-*Svpat* into pBAD-Myc/His-A, generating plasmid pBAD-*Svpat*. Cells of *E. coli* TOP10 bearing pBAD-*PparsN1* or pBAD-*Svpat* were grown in LB medium with shaking at 37 °C. At an A_600nm_ of 0.5–0.6, L-arabinose was added as an inducer at a final concentration of 0.2% (w/v). After 5 h, the cells were harvested and stored at −80 °C until use. The frozen cells were thawed and washed once with and resuspended in buffer A (50 mM morpholinopropane-1-sulfonic acid, pH 7.5, containing 20 mM imidazole, 0.5 M NaCl and 20% (v/v) glycerol) (5 ml per gram of wet cells). The cells were lysed by a one-time passage through a French pressure cell at 20,000 psi and immediately mixed with 2.5 µl per g of wet cell of diisopropylfluorophosphate. The cell lysate was centrifuged at 40,000 rpm using a T865 rotor (Thermo Fisher Scientific, Waltham, MA, USA) for 60 min at 4 °C. The supernatant solution was applied onto a Ni-NTA column (QIAGEN Sciences, Hilden, Germany) at a flow rate of 1.0 ml/min and washed with 20 column volumes (100 ml) of buffer A. Bound protein was eluted with buffer A containing 0.2 M imidazole, and the purity was assessed by sodium dodecyl sulfate polyacrylamide gel electrophoresis^40^. Protein concentrations were estimated by the Bradford assay by using bovine serum albumin as a standard. Fractions containing the protein were pooled and concentrated using a 10 kDa Amicon Ultra centrifugal filter (EMD Millipore, Billerica, MA, USA). The concentrated protein was rapidly frozen and stored at −80 °C until use.

### Glutamine synthetase assays

The activity of glutamine synthetase from *E. coli* was measured using a coupled assay that determines formation of the product ADP to oxidization of NADH^41^. The 1 ml reaction mixture contained 34 mM imidazole, 9 mM ATP, 1 mM phosphoenolpyruvate, 60 mM magnesium chloride, 19 mM potassium chloride, 45 mM ammonium chloride, 0.25 mM NADH, 13 to 20 units of l-lactic dehydrogenase and 8–14 units of pyruvate kinase. The reaction was initiated by addition of glutamine synthetase at 0.2 nM, final concentration. The decrease in *A*_340nm_ was measured at 37 °C, and oxidation of NADH to NAD^+^ was quantified using an extinction coefficient 6230 M^−1^cm^−1^. The assays were performed with concentrations of l-glutamate from 2 to 100 mM. Inhibition constants (*K*_i_) for AST and L-PPT were determined from the apparent *K*_m_ of glutamine synthetase calculated with three different concentrations of inhibitor. Activities were corrected with the values from control assays without enzyme. Kinetic constants were calculated using Sigma Plot (Systat Software, Inc., Sun Jose, CA).

### *N*-acetyltransferase assays

The enzymatic activity of purified PpArsN1 was measured from the rate of 5,5’-dithio-bis-2-nitrobenzoic acid reduction as described previously with minor modifications^42^. The reactions were carried out in 20 mM Tris-HCl (pH 7.4), 1 mM ethylenediaminetetraacetic acid, 0.33 mM 5,5′-dithio-bis-2-nitrobenzoic acid, 0.2 mM acetyl coenzyme A (AcCoA) with 50 µM AST, PPT or MSO at 37 °C. The reactions were initiated by addition of AcCoA, and the linear increase in A_412nm_ was measured over the first 2 min. The specific activity was determined using the molar extinction coefficient of 2-nitro-5-benzoatic acid (14,150 M^−1^ cm^−1^)^42^. Activities were corrected with the values from control assays without enzyme. The kinetics of PpArsN1 and SvPAT for PPT and AST were determined over a concentration range between 1 µM and 2 mM using 0.2 µM enzyme. Kinetic constants were calculated from a fit of the data to the Michaelis-Menten equation^43^ using SigmaPlot.

### Bacterial-resistance assays

Middlebrook 7H9 broth (Difco Laboratories Inc., Detroit, MI, USA) supplemented with 5 g bovine serum albumin, 2 g dextrose, 0.85 g NaCl and 0.05% tween 80 (Fisher Scientific International Inc., Pittsburg, PA, USA) was used to culture *M. bovis* BCG. Mycobacterial cells were inoculated at a density of 1.0 × 10^5^ CFU/ml and horizontally cultured in the presence or absence of the indicated concentrations of AST, L-PPT or L-MSO in an incubator humidified at 37 °C under 5% CO_2_ for up to 4 weeks. Viable cells in each culture were determined by *A*_600nm_. All other bacterial strains were grown in LB medium overnight, following which the cells were centrifuged, washed with and resuspended in M9 medium^39^ to an *A*_600nm_ of 0.04−0.06, with or without the indicated concentrations of As(III), MAs(III), AST, L-PPT or L-MSO. M9 medium was supplemented with 0.2% (w/v) citrate and 20 µg/ml uracil for *P. putida* strains, while M9 medium supplemented with 0.2% (w/v) glucose was used for the other bacterial strains. 0.1 mg/ml ampicillin and 0.2% (w/v) arabinose as inducer were added to cultures of *E. coli*, as required. Resistance was determined from the A_600nm_ after 24 h. *E. coli* and *B. megaterium* were grown at 37 °C. Other bacterial strains were cultured at 30 °C.

### Cytotoxicity assays

Human acute monocytic leukemia THP-1 cells (ATCC TIB-202^TM^) were seeded in a 24-well plate (Nalge Nunc International, Rochester, NY, USA) with 300 µl of RPMI-1640 medium (Lonza, Basel, Switzerland) supplemented with 10% (v/v) fetal bovine serum and 0.05 mM 2-mercaptoethaol at a density of 1.0 × 10^5^ cells/well and cultured in a 5% CO_2_ humidified incubator at 37 °C. After 24 h, THP-1 cells were further cultured in the presence or absence of the indicated concentrations of AST or As(III) for another 24 h, following which viability of cells was determined by a 3-(4,5-dimethylthiazol-2-yl) 2,5-diphenyltetrazolium bromide assay^44^. 3-(4,5-dimethylthiazol-2-yl) 2,5-diphenyltetrazolium bromide was added to each well at a final concentration of 0.5 mM and the cultures were incubated for 3 h. The plate was then centrifuged at 400 × *g*, the cell pellets were lysed with 300 µl of dimethyl sulfoxide to dissolve 3-(4,5-dimethylthiazol-2-yl) 2,5-diphenyltetrazolium bromide formazan. Cell viability was estimated from A_570nm._

### ArsN distribution and phylogenetic analysis

The prevalence of *arsN* (*arsN1* and *arsN2*, see Results) genes in *ars* operons was analyzed in representative organisms. GenBank accession numbers of the following bacterial genomes are given in parentheses. *P. putida* KT2440 (AE015451), *Bacillus sp*. GZT (LVVJ00000000), *Meiothermus chliarophilus* DSM 9957 (AUQW00000000), *Acidovorax* sp. CF316 (AKJX00000000), *Porphyrobacter mercurialis* (JTDN00000000), *Pseudomonas syringae* pv*. syringae* B728a (CP000075), *Inquilinus limosus* DSM 16000 (AUHM00000000), *Sphingopyxis* sp. KK2 (LYVN00000000), *Sphingomonas yabuuchiae* (LDTF00000000), *Pelomonas* sp. KK5 (LYVQ00000000), *Burkholderia lata* (CP000150), *Rhodobacter sphaeroides* ATCC17025 (CP000661), *Rubrobacter xylaniophilus* DSM 9941 (CP000386), *Mumia flava* (JTDJ00000000), *Roseiflexus castenholzii* DSM 13941 (CP000804), *Ralstonia pickettii* 12 J (AAWK00000000), *Rhizobium leguminosarum* bv. *viciae* 3841 (AM236080), *Burkholderia phytofirmans* PsJN (AAUH00000000), *Spirosoma panaciterrae* DSM 21099 (ARFA00000000), *Oxalobacteraceae bacterium* AB_14 (ARMC00000000), *Geobacillus kaustophilus* HTA426 (BA000043), *Paenibacillus stellifer* DSM 14472 (NZ_CP009286), *Thermoflavimicrobium dichotomicum* DSM 44778 (NZ_FORR01000002), *Deinococcus aquatilis* DSM 23025 (NZ_KB899704), *Meiothermus terrae* DSM 26712 (NZ_QXDL01000023). Multiple alignment of the sequences of putative *N*-acetyltransferase orthologs was performed using T-Coffee^45^ and BoxShade^46^. *N*-acetyltransferase sequences distributed in *ars* operons^20,21^ were defined as ArsN [*P. putida* KT2440 (WP_010952945), *Bacillus* sp. GZT (WP_062922891), *M. chliarophilus* DSM 9957 (WP_027893731, WP_027893733), *Acidovorax* sp. CF316 (WP_007857208), *P. mercurialis* (WP_039093634), *P. syringae* pv. *syringae* B728a (YP_234588), *I. limosus* DSM 16000 (WP_026871525), *Sphingopyxis* sp. KK2 (WP_077145629), *S. yabuuchiae* (WP_058746515, WP_058746517), *Pelomonas* sp. KK5 (WP_077035561), *B. lata* (WP_011349260), *R. sphaeroides* ATCC 17025 (WP_011908437), *R. xylanophilus* DSM 9941 (WP_011565797), *M. flava* (KHL15495), *R. castenholzii* DSM 13941 (WP_012120818), *R. pickettii* 12 J (WP_012429982), *R. leguminosarum* bv. *viciae* 3841 (WP_011652407), *B. phytofirmans* PsJN (WP_012431288), *S. panaciterrae* DSM 21099 (WP_020601039) and *O. bacterium* AB_14 (WP_020703167), *G. kaustophilus* HTA426 (BAD74878), *M. terrae* DSM 26712 (WP_119314079), *P. stellifer* DSM 14472 (WP_038700913), *T. dichotomicum* DSM 44778 (WP_093227883) and *D. aquatilis* DSM 23025 (WP_019009361)]. *N*-acetyltransferase sequences distributed in phosphinothricin tripeptide biosynthesis gene clusters^27^ were defined as phosphinothricin *N*-acetyltransferase (PAT) [*Streptomyces hygroscopicus* (P16426), *Streptomyces viridochromogenes* (WP_003988626), *Kitasatospora phosalacinea* NRRL B-16230 (KP185121) and *Actinobacteria bacterium* OK074 (WP_082414639)]. *N*-acetyltransferases with higher selectivity for PPT compared to MSO^47-49^ are also defined as PAT [*Streptomyces coelicolor* A3(2) (CAB90987), *Rhodococcus* sp. YM12 (JQ398613) and *Nocardia* sp. AB2253 (BAG06876)]. *N*-acetyltransferases with higher selectivity on MSO compared to PPT^21,47,50,51^ are defined as methionine sulfoximine *N*-acetyltransferase (MAT) [*E. coli* K-12 (AAC74530), *Salmonella enterica Typhimurium* str. LT2 (NP_460549), *P. putida* KT2440 (WP_010955452) and *Pseudomonas aeruginosa* PAO1 (AAG08251), *Acinetobacter* sp. ADP1 (Q6FBS8)]. *N*-acetyltransferases that have similar activity on both PPT and MSO^47^ are also included [*Geobacillus kaustophilus* HTA426 (BAD77205), *Bacillus subtilis* RO-NN-1 (AEP92705), *Paraburkholderia xenovorans* LB400 (ABE30708, ABE34181), *Staphylococcus aureus* USA300_FPR3757 (ABD22256) and *Deinococcus radiodurans* R1 (AAF10750)]. GenBank accession numbers of *N*-acetyltransferase orthologs are given in parentheses. Phylogenetic analysis was performed to infer the evolutionary relationship among the sequences of ArsN1, PAT and/or MAT from various organisms. The phylogenetic tree was constructed using the Neighbor-Joining method using MEGA X^52^. The statistical significance of the branch pattern was estimated from a 1000 bootstrap^53^.

### Crystallization and structure determination

Initial crystallization screening was performed as described previously^54^ by the sitting-drop vapor-diffusion method^55^ using a variety of crystal screens from Hampton Research (Aliso Viejo, CA, USA), Emerald BioSystems, Inc. (Bainbridge Island, WA, USA) and Jena Bioscience GmbH (Jena, Germany) in 96-well plates (Corning Inc., Corning, NY, USA) at 293 K. Crystalline precipitates were obtained at 0.2 M sodium acetate, 0.1 M Tris-HCl, pH 8.5, and 30% (w/v) PEG 4000. Diffraction quality crystals were grown using the vapor diffusion hanging drop method in 24-well Linbro plates. The reservoir solution (0.3 ml) consisted of 0.2 M sodium acetate, 0.1 M Tris-HCl and 20% (w/v) PEG 6000, and the hanging drop contained 2 µl of 20 mg/ml of purified PpArsN1, 2 µl reservoir solution and 1 µl of 0.1 M ATP. Rod-shaped crystals, with approximate dimensions of 0.1 × 0.05 × 0.05 mm, were obtained within a few weeks. The PpArsN1-AST complex was prepared by adding 0.5 ml of 4.0 mM AST to 0.5 ml of 1 mM protein. PpArsN1-AST crystals were grown using vapor diffusion hanging drop method. The hanging drop contained 2 µl of PpArsN1-AST complex and 2 µl of reservoir solution. The reservoir contained 1.5 M sodium formate and 0.1 M sodium acetate with pH 4.5. Thin plate-like crystals were obtained within a week. The PpArsN1-PPT complex was prepared by adding 0.5 ml of 25 mM L-PPT to 0.5 ml of 1 mM protein. The PpArsN1-PPT crystals were also grown using the same method and crystallization condition as used for the PpArsN1-AST crystals. The crystals were harvested from the hanging drop using a cryoLoop, flash-frozen in liquid nitrogen at 100 K and stored in liquid nitrogen. Ethylene glycol (20%, v/v) was used as cryoprotectant. X-ray data were collected on beamline 22ID at the Advanced Photon Source), Argonne National Laboratory, using a MAR300HS detector. The crystal-to-detector distance was 180 mm, and 180 images for PpArsN1 crystal, 240 and 360 images for PpArsN1-AST and PpArsN1-PPT crystals, respectively, were collected with 1° oscillations. The PpArsN1 diffraction data were indexed and scaled using KYLIN^56^ and PpArsN1-AST and PpArsN1-PPT data were indexed and scaled using HKL2000^57^. The data processing statistics are shown in Table 2. The PpArsN1 crystal diffracted to 2.16 Å resolution. The crystal belongs to space group P4_3_2_1_2 with cell dimensions *a* = *b* = 67.02 Å, *c* = 206.74 Å. The Matthews coefficient of 2.48 indicates that there are two molecules in the asymmetric unit with 50.5% solvent. An initial homology model was constructed by molecular replacement with an acetyltransferase from *P. aeruginosa* PA01 (PDB ID: 1YVO as a template with 32.3 % identity) using SWISS-MODEL^58^. Molecular replacement was done using PHASER^59^ in the CCP4 suite^60^. The initial R and *R*_free_ were 35.0 and 40.0%, respectively. The structure was refined using PHENIX^61^. The C-terminal extended residues were fitted in electron density using COOT^62^. Water molecules were added at appropriate positions and refined. The final R and R_free_ are 23.7% and 26.6%, respectively. The PpArsN1-AST crystal diffracted to 2.19 Å resolution and indexed with C121 space group with cell dimensions *a* = 185.27, *b* = 141.74, *c* = 54.55 Å and *β* = 90.6°. The Matthews coefficient of 2.54 indicates that there are six molecules in the asymmetric unit with 51.6% solvent. The PpArsN1-apo structure was used as a model for molecular replacement. There are positive electron densities at the 9.0 and 16.0 σ level near Arg77 in molecule A and B, respectively (Supplementary Figure 9). The density was fitted with the L-enantiomer of AST, and the anomalous difference map confirmed the presence of arsenic. The PpArsN1-PPT crystal diffracted to 2.66 Å resolution and indexed with P12_1_1 space group with cell dimensions *a* = 53.84, *b* = 142.69, *c* = 178.31 Å and *β* = 89.9°. The Matthews coefficient of 2.45 indicates that there are twelve molecules in the asymmetric unit with 49.8% solvent. The PpArsN1-apo structure was used as model for molecular replacement. There are positive electron densities between 6.5 and 9.0 σ level near Arg77 in molecule A–D, G–J (Supplementary Figure 10). The density was fitted with two L-PPT molecules. The structure were refined using REFMAC5^63^ in the CCP4 suite^60^. The simulated annealing refinement was done using PHENIX. The structure factor and coordinates were deposited to the Worldwide Protein Data Bank (wwPDB, accession IDs: 5JTF (PpArsN1), 5WPH (PpArsN1-AST) and 6M7G (PpArsN1-PPT)). The molecules were drawn with PyMol (Version 1.8 Schrödinger, LLC). Docking was performed using AutoDockTools and AutoDock4^64^.

**Table 2:** Data collection and refinement statistics

	PpArsN1^a^	PpArsN1-AST^a^	PpArsN1-PPT^a^
*Data collection*			
Space group	P4_3_2_1_2	C121	P12_1_1
*Cell dimensions*			
*a, b, c* (Å)	67.02, 67.02, 206.74	185.27, 141.74, 54.55	53.84, 142.69, 178.31
*α, β, ϒ* (°)	90.0, 90.0, 90.0	90.0, 90.6, 90.0	90, 89.9, 90.0
Resolution (Å)	30.64–2.16 (2.20–2.16)^b^	50.00–2.19 (2.23–2.19)^b^	39.91–2.66 (2.75–2.66)^b^
*R*_merge_	0.078 (0.342)^b^	0.150 (0.654)^b^	0.157 (1.144)^b^
*I/* *σ* *I*	6.9 (2.9)^b^	12.4 (1.9)^b^	11.0 (1.9)^b^
Completeness (%)	97.6 (90.8)^b^	99.4 (95.7)^b^	97.0 (97.2)^b^
Redundancy	5.16 (4.31)^b^	7.3 (4.4)^b^	4.2 (3.9)^b^
*Refinement*			
Resolution (Å)	30.64–2.16 (2.24–2.16)^b^	49.02–2.19 (2.24–2.19)^b^	39.91–2.66 (2.69–2.66)^b^
No. of reflections	25,795	67,549	70,774
*R*_work_/*R*_free_	0.237/0.266	0.180/0.232	0.224/0.272
*No. of atoms*			
Protein	2681	8443	15,829
Ligand/ion	-	26	89
Water	104	874	222
*B*-*factors*			
Protein	34.95	29.97	39.49
Ligand/ion	-	41.66	55.00
Water	38.10	35.12	37.89
*R.m.s. deviations*			
Bond lengths (Å)	0.004	0.013	0.004
Bond angles (˚)	0.905	1.477	0.880

^a^Each structure was refined from a single data set from an independent protein crystal

^b^Values in parentheses are for highest resolution shell

### Statistics

Assays of glutamine synthetase, *N*-acetyltransferase, bacterial resistance and cytotoxicity were repeated at least three times. The data are presented as the mean ± standard error (SE). No other statistical tests were performed.

### Reporting summary

Further information on experimental design is available in the Nature Research Reporting Summary linked to this article.

## Supplementary information

Supplementary Information

Supplementary Data 1

Description of Additional Supplementary Files

Reporting Summary

## Data Availability

Protein structural data have been deposited in the wwPDB under accession IDs 5JTF (PpArsN1), 5WPH (PpArsN1-AST) and 6M7G (PpArsN1-PPT). The source data used to generate the Figs. 2 and 5 are presented as Supplementary Data 1. Other data that support the findings of the current study are available from the corresponding authors on reasonable request.
