# Supplementary material for: Arsinothricin, an arsenic-containing non-proteinogenic amino acid analog of glutamate, is a broad-spectrum antibiotic
Source: Commun Biol. 2019 Apr 15;2:131. doi: 10.1038/s42003-019-0365-y (PMC6465285; doi:10.1038/s42003-019-0365-y)
Supplement: Supplementary file 1 — Supplementary Information [file 42003_2019_365_MOESM1_ESM.pdf]

Supplementary Figure 1.

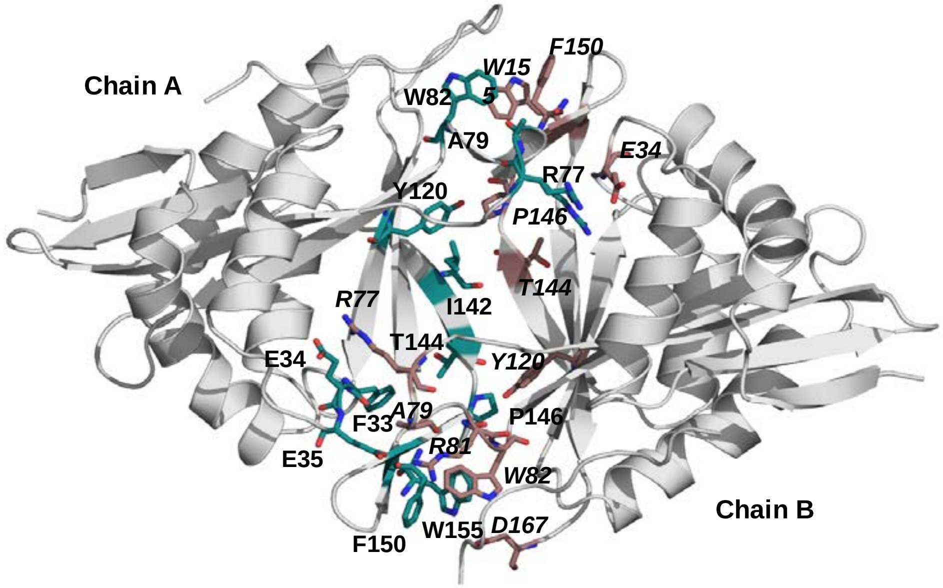

Crystallographic analysis of the PpArsN1 dimer. The crystallographic asymmetric unit with Chains A and B is shown. The interfacial residues are shown in stick representation. Chain A residues are colored in teal, and Chain B residues are shown in brown and are labeled in italics.

Supplementary Figure 2.

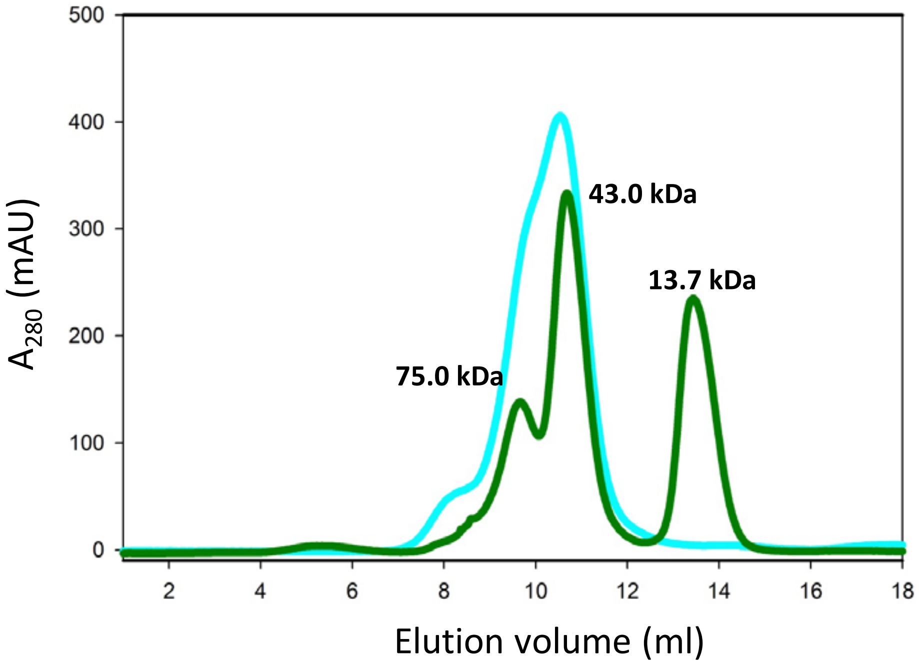

PpArsN1 is a dimer in solution. The oligomerization state of purified protein was analyzed by gel filtration. Purified PpArsN1 was chromatographed through Superdex75 in a 10/300 GL column. Elution of PpArsN1 is shown in cyan, and a mixture of proteins of known molecular mass is shown in green.

Supplementary Figure 3.

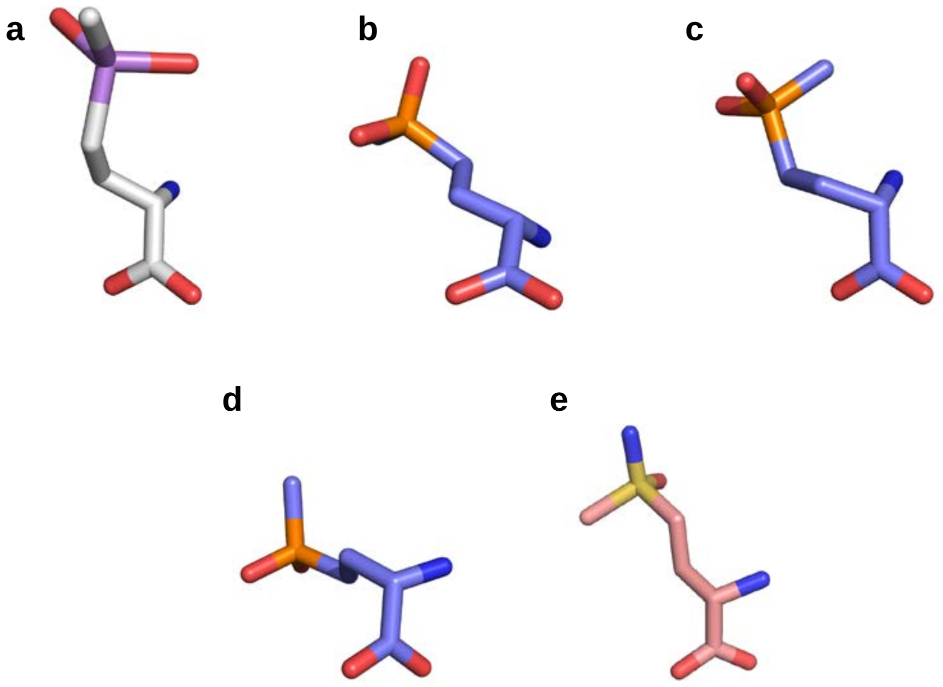

The L-enantiomer of AST is bound to PpArsN. Comparison of ArsN1-bound AST (**a**), PPT-1 (**b**) and PPT-2 (**c**) with ShPAT-bound L-PPT (PDB ID: 5T7E) (**d**) and PaMAT-bound L-MSO (PDB ID: 2J8R) (**e**) confirms that PpArsN1-bound AST is the L-enantiomer. PaMAT: Methionine sulfoximine *N*-acetyltransferase from *Pseudomonas aeruginosa* PAO1 (GenBank accession number: AAG08251).

Supplementary Figure 4.

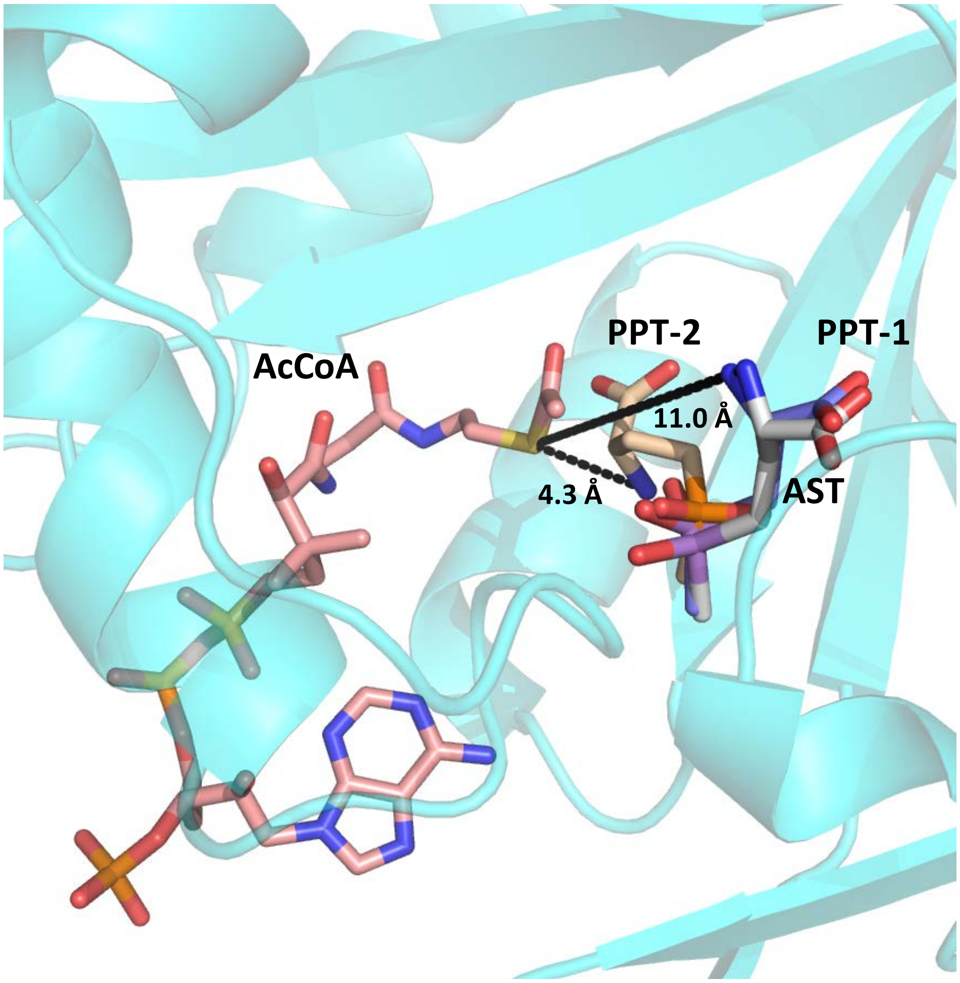

Model of AcCoA- and substrate-bound PpArsN1. The AcCoA binding site was predicted by docking AcCoA with the structure of PpArsN1 using Autodock4. The distance between the amino group of AST and that of PPT-1 and the sulfur atom of AcCoA is approximately 11.0 Å, which is too far for N-acetylation. The distance between the amino group of PPT-2 and the sulfur atom of AcCoA is 4.3 Å, which is within the possible distance for *N*-acetylation. We propose that the substrates move into position for *N*-acetylation by conformational changes during the catalytic cycle.

Supplementary Figure 5.

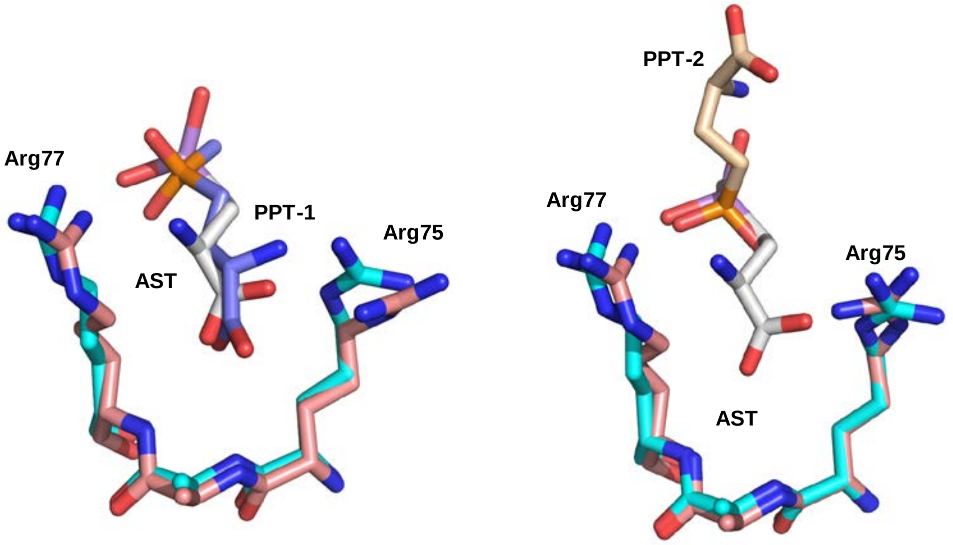

Superposition of residues Arg77 and Arg75 of AST and PPT bound PpArsN1 structures. A portion of the AST binding site in PpArsN1-AST (cyan) is superimposed with that of the PpArsN1-PPT structures (salmon). Left and right cartoons depict Chain A and Chain B, respectively.

Supplementary Figure 6.

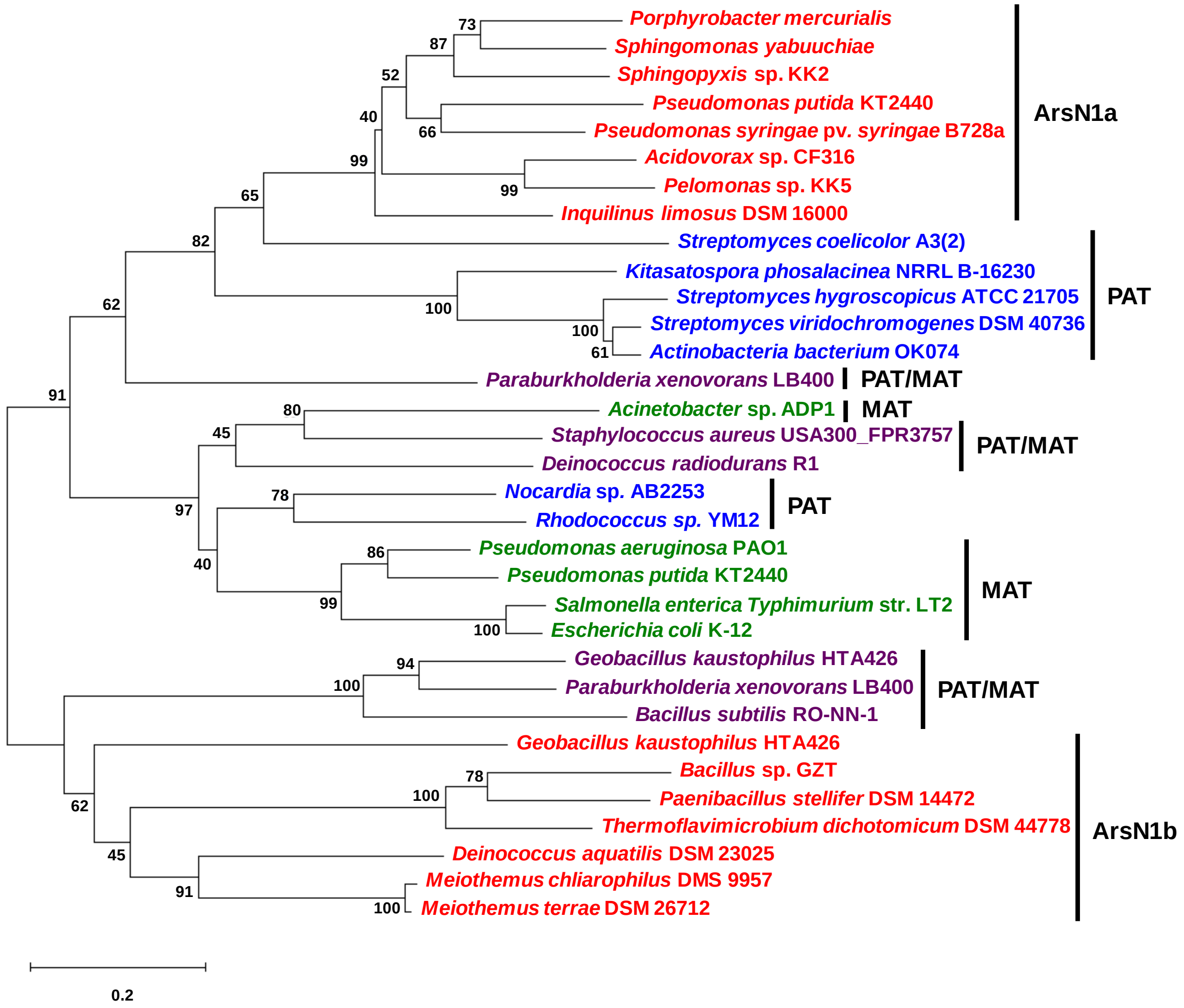

Phylogeny of *N*-acetyltransferase genes for resistance to AST, PPT and MSO. The neighbor-joining phylogenetic tree shows the evolutionary relationships of ArsN1, PAT and MAT. ArsN1 (highlighted in red), PAT (blue) and MAT (green) are defined as described in *Methods*. *N*-acetyltransferases that have similar activity with both PPT and MSO are labeled as PAT/MAT (purple). ArsN1 genes are further sorted into two clades (ArsN1a and ArsN1b). Bootstrap values calculated for 1,000 subsets (%) are indicated on each branch. GenBank accession numbers of bacterial genomes are given in Methods. The scale bar represents 20% sequence dissimilarity.

Supplementary Figure 7.

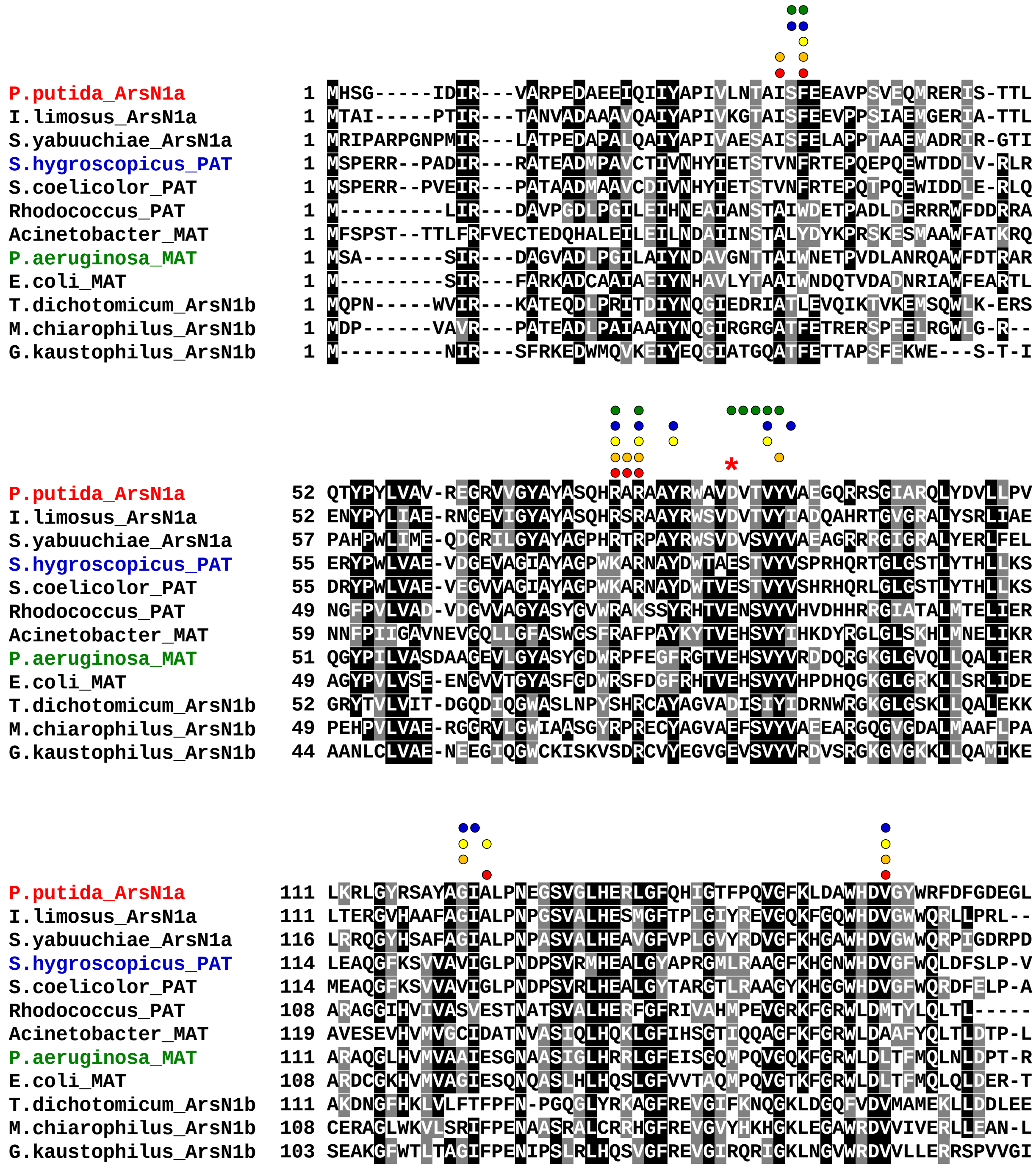

Multiple protein sequence alignment of *N*-acetyltransferase orthologs of ArsN1, PAT and MAT. Circles of red, orange and yellow indicate the substrate-binding residues for AST, PPT-1 and PPT-2, respectively, in *P. putida* ArsN1 (red letters). Circles of blue and green indicate the

PPT-binding residues in *S. hygroscopicus* PAT (blue letters) and *P. aeruginosa* MAT (green letters), respectively. Asp85 in PpArsN1 is replaced by Glu residue in both PPT N-acetyltransferase. The position of the conserved catalytic residue Glu88 in ShPAT that acts as a general base is highlighted by a red asterisk. C-terminal sequences that are poorly conserved are not shown.

Supplementary Figure 8.

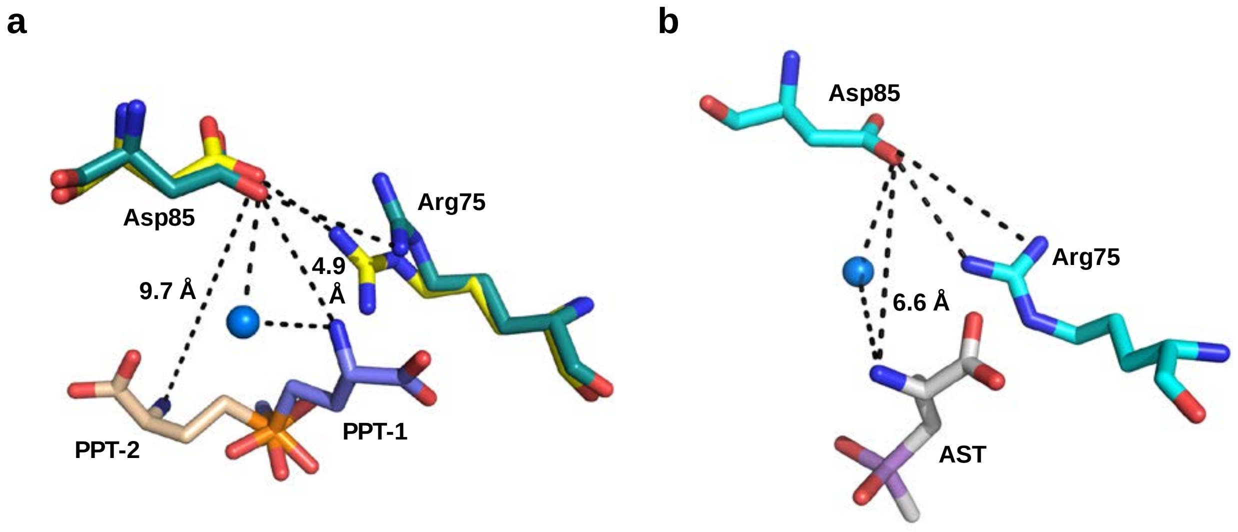

Interaction of PPT (a) and AST (b) with Asp85 via a water molecule. (a) The distance between the amino group of L-PPT and the side chain of Asp85, a predicted catalytic residue, is 4.9 Å and 9.7 Å in the PPT-1 and PPT-2 conformations, respectively. The amino group of L-PPT interacts Asp85 through water molecule in the PPT-1 conformation but not in the PPT-2 conformation. Residues from PpArsN1 in the PPT-1 and PPT-2 conformations are shown in yellow and teal blue, respectively. **(b)** The distance between the amino group of L-AST and the side chain of Asp85 is 6.6 Å. As seen in the PPT-1 conformation, the amino group of L-AST form a water molecule interaction with the side chain of Asp85. Water molecules are in blue spheres.

Supplementary Figure 9.

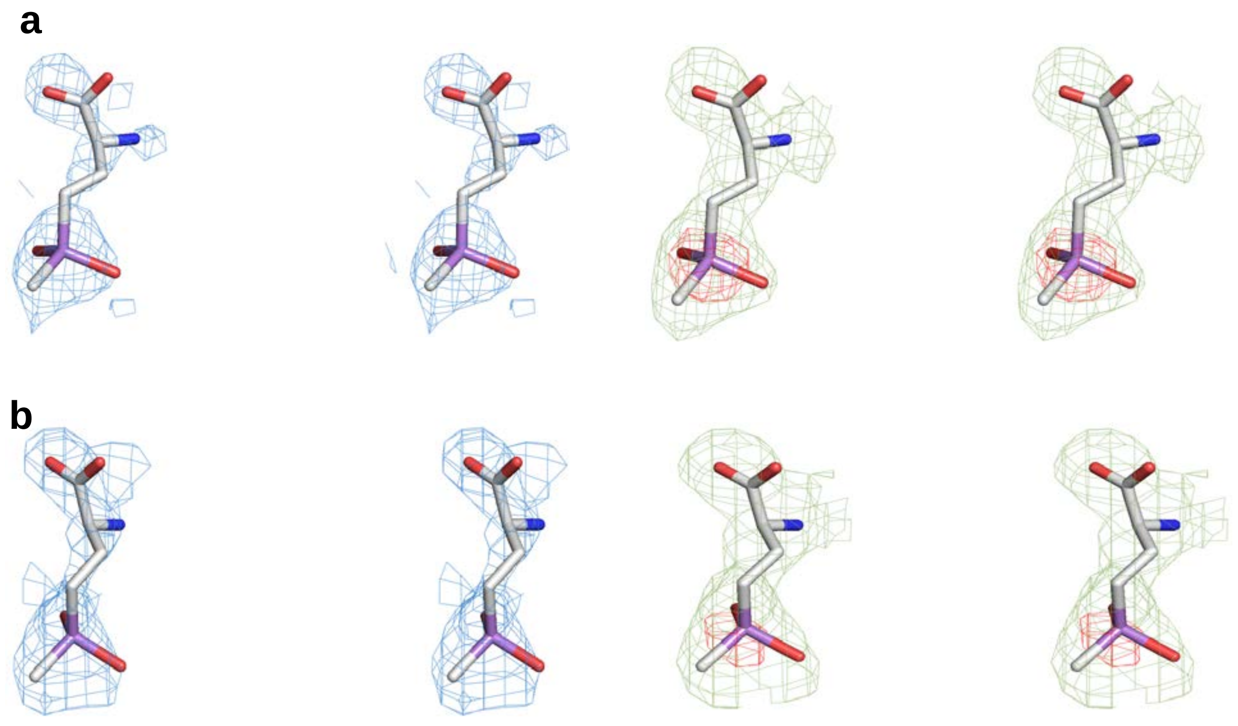

Stereo view of the electron density of AST in the PpArsN1 structure. a. AST in chain A. **b.** AST in chain B. Electron density (2Fo-Fc) map for AST contoured at the 1.0 σ level (blue) omit map (Fo-Fc) at 2.0 σ (green) and anomalous difference map at the 3.0 σ level (red) of AST.

Supplementary Figure 10.

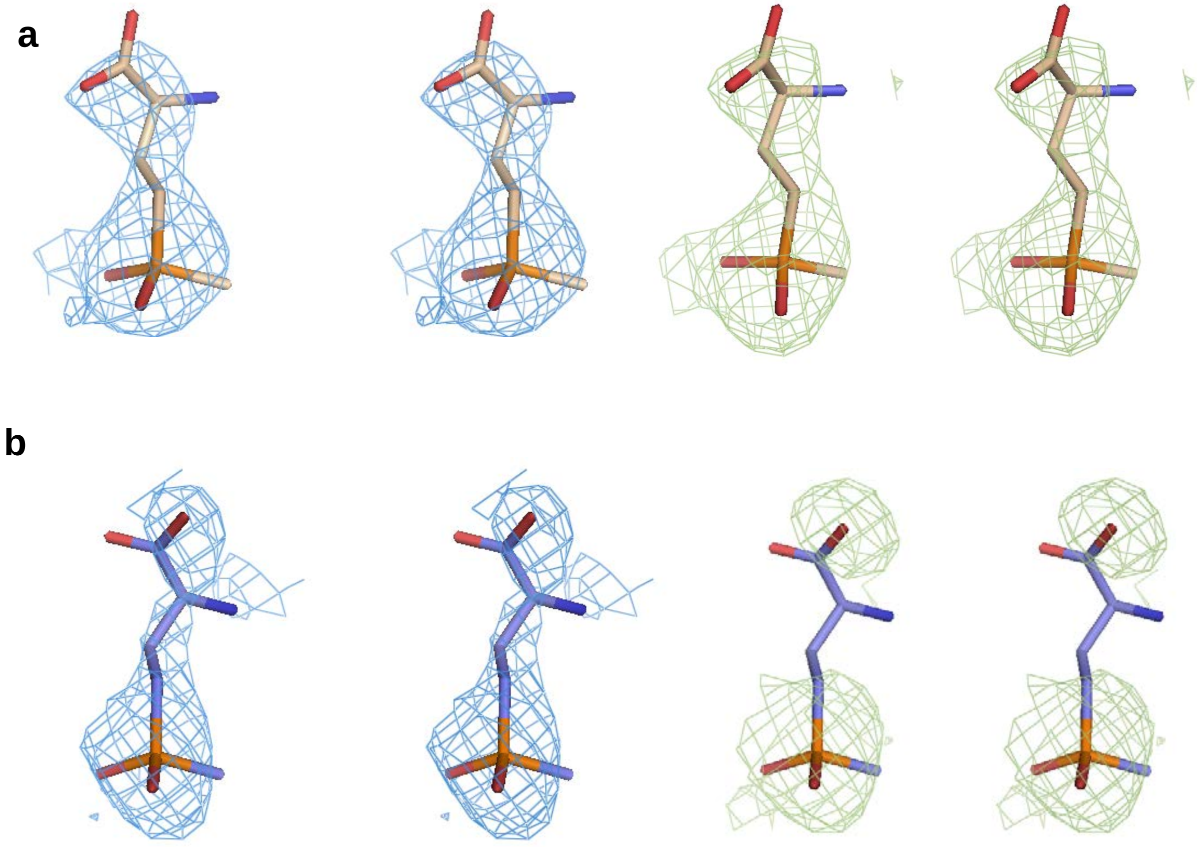

Stereo view of electron density of PT in the PpArsN1 structure. a. PPT-2 (conformation 2). **b.** PPT-1 (conformation 1). Electron density (2Fo-Fc) map for PPT contoured at the 1.0 σ level (blue); Omit map (Fo-Fc) at 2.0 σ level of PPT (green).
