# Supplementary material for: Arsinothricin, an arsenic-containing non-proteinogenic amino acid analog of glutamate, is a broad-spectrum antibiotic
Source: Commun Biol. 2019 Apr 15;2:131. doi: 10.1038/s42003-019-0365-y (PMC6465285; doi:10.1038/s42003-019-0365-y)
Supplement: Supplementary file 3 — Description of Additional Supplementary Files [file 42003_2019_365_MOESM3_ESM.docx]

**Description of Supplementary Data 1**

**File Name**: Supplementary Data 1

**Description**: The source data underlying the graphs and charts presented in Figures 2 and 5 are shown.
